# Increased sensitivity of next generation sequencing-based expression profiling after globin reduction in human blood RNA

**DOI:** 10.1186/1471-2164-13-28

**Published:** 2012-01-18

**Authors:** Anastasios Mastrokolias, Johan T den Dunnen, GertJan B van Ommen, Peter AC 't Hoen, Willeke MC van Roon-Mom

**Affiliations:** 1 Center for Human and Clinical Genetics, Leiden University Medical Center, Einthovenweg 20, 2333ZC, Leiden, The Netherlands; 2 Leiden Genome Technology Center, Leiden University Medical Center, Einthovenweg 20, 2333ZC Leiden, The Netherlands

## Abstract

**Background:**

Transcriptome analysis is of great interest in clinical research, where significant differences between individuals can be translated into biomarkers of disease. Although next generation sequencing provides robust, comparable and highly informative expression profiling data, with several million of tags per blood sample, reticulocyte globin transcripts can constitute up to 76% of total mRNA compromising the detection of low abundant transcripts. We have removed globin transcripts from 6 human whole blood RNA samples with a human globin reduction kit and compared them with the same non-reduced samples using deep Serial Analysis of Gene Expression.

**Results:**

Globin tags comprised 52-76% of total tags in our samples. Out of 21,633 genes only 87 genes were detected at significantly lower levels in the globin reduced samples. In contrast, 11,338 genes were detected at significantly higher levels in the globin reduced samples. Removing globin transcripts allowed us to also identify 2112 genes that could not be detected in the non-globin reduced samples, with roles in cell surface receptor signal transduction, G-protein coupled receptor protein signalling pathways and neurological processes.

**Conclusions:**

The reduction of globin transcripts in whole blood samples constitutes a reproducible and reliable method that can enrich data obtained from next generation sequencing-based expression profiling.

## Background

Transcriptomics technologies have successfully been used for biomarker discovery and the study of physiological and pathophysiological mechanisms [1]. Recently, advances in sequencing technology have allowed for direct identification of transcript specific sequences (tags) that are digitally counted, and the analysis of differences in gene expression with unprecedented accuracy [2]. One specific application of next generation gene expression analysis is DeepSAGE, a tag sequencing method on the Illumina high-throughput sequencing platform that is analogous to LongSAGE [3]. Such sequencing-based technologies offer distinct advantages over expression micro-arrays, such as a higher dynamic range, the increased power for detection of low abundance transcripts, and the detection of novel transcripts and transcript variants [4,5]. Furthermore, next generation sequencing technologies show less variation between different study sites than microarray technology and are not content-limited [6].

Transcriptome analysis of peripheral blood is of great interest for clinical research; where significant differences between samples obtained in a minimally invasive and cost-effective manner can be translated into gene signatures of disease stage, drug response and toxicity [7]. Blood comes into contact with almost every tissue and organ of the human body and due to its cellular composition it can reflect both physiological and pathogenic stimuli. Gene expression differences in peripheral whole blood have been used to determine signatures related to acute myeloid leukaemia [8], but also in neuropsychiatric disorders and Huntington's disease where significant correlation was found between blood and brain gene expression [9,10]. Furthermore disease signatures can be robust across tissues and experiments and in a large meta-analysis study performed by Dudley and colleagues, it was demonstrated that gene expression profiles in various tissues from the same disease were more similar than gene expression profiles from identical tissues from different diseases [11]. Furthermore, 80% of genes expressed in peripheral blood cells are shared with other important tissues [12]. These results suggest that transcriptome analysis of the blood is often informative even when the originating pathology stems from a different tissue.

Blood is composed of three main cell types. The main component is red blood cells (95%), including the progenitor erythrocytes called reticulocytes, followed by platelets (5%) and white blood cells (< 1%). While white blood cells make up the minority, they are the most informative and in the past, many studies have been performed on the isolated white blood cell fraction. However, white blood cell isolation kits can induce a technical bias that can confound the original expression profile of the samples and delayed sample processing can affect gene expression profiles [13,14]. This means that white blood cell separation protocols require quick and accurate processing, which can be difficult in a clinical setting. The expression profile of a sample is better preserved by a whole blood collection method, like the PAX gene blood RNA system [15]. Another advantage of this system is that samples can be frozen for up to 2 years without affecting the expression profile [16]. However, cell sorting and counting is not possible because blood cells are lysed directly after collection. Therefore, abundant transcripts in abundant cell types may conceal the more interesting less abundant transcripts from less abundant cell types. Particularly, the presence of globin transcripts originating from reticulocytes in whole blood samples may limit the sensitivity of gene expression profiling experiments [14], since globin transcripts can constitute up to 70% of the total whole blood mRNA population [17]. While in microarray experiments the presence of globin transcripts may reduce the amount of fluorescent label available for other transcripts but otherwise just results in a saturated spot on the microarray, the high abundance of globin transcripts is more of a concern in sequencing-based expression profiling studies. Since measuring absolute abundance, globin transcripts will be sequenced over and over again, while limiting the coverage of other transcripts.

To deal with the abundance of globin transcripts in whole blood mRNA, several globin reduction protocols have been successfully used in gene expression studies [18-21]. The removal of alpha and beta globin mRNA can be achieved by selective hybridization of biotinylated globin sequence specific oligos with the globin transcripts and depleting them from the total mRNA population through magnetic beads [17]. Another approach developed by Affymetrix and PreAnalytiX utilizes 3' specific PNAs to inhibit reverse transcription during cDNA synthesis. Alternatively, a high abundance transcript depletion protocol adopts the properties of the Kamtchaka crab duplex specific nuclease to selectively reduce the most abundant RNA transcripts [22].

Our aim in the present study is to investigate the effect of globin transcript reduction on the specificity and sensitivity of digital gene expression profiling (deep SAGE).

## Results

### Globin transcripts can successfully be reduced in whole blood samples

**Figure 1:**
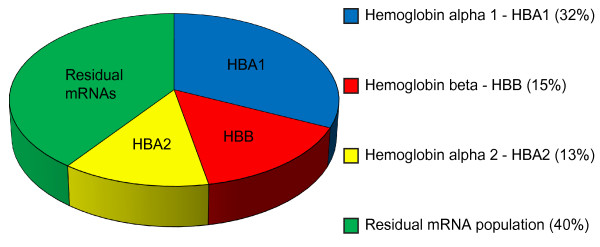
**Transcript abundance in the non-globin reduced samples**. Pie chart showing the most abundant transcripts in whole blood (average of 6 samples). Hemoglobin alpha 1 (HBA1), hemoglobin beta (HBB) and hemoglobin alpha 2 (HBA2) comprise the majority of transcripts.

To investigate whether the RNA quality was affected by globin reduction, the RNA Integrity Number (RIN) value was determined for each sample (summarized in Table 1) with a RIN value of 10 representing the highest RNA quality. On average, the RIN value was slightly lower in the globin-reduced samples compared to the non-reduced samples indicating a minor reduction in RNA quality. Additionally, we measured RNA concentrations before and after globin reduction and observed an RNA yield loss of 5-9%. General sequencing and alignment statistics are given in Table 1. To verify if globin reduction was successful, the percentage of globin transcripts was determined in the reduced and non-reduced samples. The most abundant transcripts in the non-reduced samples corresponded to globin alpha1 (*HBA1*), globin beta (*HBB*) and globin alpha2 (*HBA2*) (Figure 1). The percentage of globin transcripts constituted on average 60% (52-76%) of total reported alignments and for the reduced samples this dropped to 0.1% - 0.4% (Table 1), demonstrating the efficiency of the globin reduction protocol. The two transcripts that were most abundant after the globin transcripts, lysozyme (*LYZ*) and ribosomal protein (*RPLP2*) transcripts were detected at higher levels in the globin-reduced samples. In order to validate the next generation sequencing results we performed qPCR for *HBA *and *HBB *for 4 non reduced and reduced samples and the results confirmed the next generation sequencing results (see Additional file 1).

**Table 1:** Sequence Statistics and RNA qualities

Samples	Non- globin reduced						Globin-reduced					
	**1**	**2**	**3**	**4**	**5**	**6**	**1**	**2**	**3**	**4**	**5**	**6**

**RIN**	8.1	7.7	7.1	6.9	7.3	8.8	7.6	7.2	6.7	6.9	7	8.2

**Tag numbers**	29.2^.^10^6^	13.2^.^10^6^	32.0^.^10^6^	11.6^.^10^6^	13.6^.^10^6^	10.1^.^10^6^	16.5^.^10^6^	28.7^.^10^6^	15.9^.^10^6^	15.3^.^10^6^	15.9^.^10^6^	14.9^.^10^6^

**Non- aligned**	1.27%	5.63%	1.43%	13.80%	5.64%	1.92%	3.64%	2.95%	3.37%	3.29%	3.45%	3.38%

**Aligned**	89.9%	84.4%	89.9%	80.1%	89.1%	91%	78.3%	78.9%	78.4%	78.9%	77.6%	77.5%

**Globin tags**	60.1%	52.1%	57.2%	69.6%	76%	71%	0.3%	0.4%	0.4%	0.2%	0.4%	0.1%

Quality of RNA depicted by RNA integrity numbers (RIN) for the 6 non-globin reduced and globin reduced samples. The number of tags, percentages of aligned and non-aligned sequences, and relative number of globin tags are shown as percentage of the total number of aligned tags.

To examine the reproducibility of globin reduction we performed a logarithmic transformation on the count data from the 6 globin reduced samples and calculated the correlation. Correlation values ranged from 0.88 to 0.94, showing good reproducibility after globin reduction (Additional file 2).

### Differential Gene Expression Differences between Globin Reduced and Non-Reduced Samples

**Figure 2:**
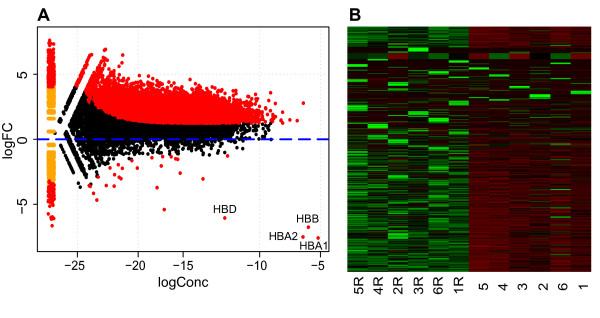
**Differential expression of non-globin reduced versus globin reduced whole blood samples**. **A**. MA-plot (log concentration on x-axis, log fold-change on y-axis) showing that the majority of differentially expressed genes (*P *value < 0.01, indicated in red) are up-regulated while the 4 globin genes showed the strongest reduction. Genes with zero expression in one of the groups are shown on the left end of the plot. *HBD*: hemoglobin delta, *HBB*: hemoglobin beta, *HBA1*: hemoglobin alpha 1, *HBA1*: hemoglobin alpha 2. *CXorf25 *has a logConc of -18.79 and a logFC of -14.3 and is not shown on this graph. **B**. Heatmap of normalised expression values of 21633 genes across the 6 non reduced and 6 reduced (R) samples where green depicts high expression and red low expression values.

To investigate if more genes could be identified after globin reduction we used the edgeR Bioconductor statistical R package to examine differential gene expression between the 6 globin reduced and 6 non-reduced samples. We identified 21,633 uniquely expressed genes in the 12 samples and 11,633 out of these 21,633 genes were detected at significantly higher levels (FDR 1%) in the globin reduced samples (Figure 2). There were 87 genes detected at significantly lower levels in the globin reduced samples. The transcripts that were the most significantly decreased (p-value) were the pseudogene CXorf25 and the 4 globin transcripts globin delta (*HBD*), hemoglobin beta (*HBB*), hemoglobin alpha 1(*HBA1*), hemoglobin alpha 2 (*HBA2*) (see Table 2). Furthermore, 82 other non-globin transcripts were significantly reduced by the globin reduction procedure (Additional File 3).

**Table 2:** Top ten most differentially expressed transcripts

Gene Description	Gene name	logConc^1 ^	logFC^2 ^	pValue	FDR
Pseudogene	CXorf25	-18.79	-14.3	2.10e-54	3.68e-50

Globin Alpha1	HBA1	-5.19	-7.59	7.48e-34	6.53e-30

Globin Alpha2	HBA2	-6.43	-7.51	2.12e-33	1.23e-29

Globin Beta	HBB	-5.99	-6.76	3.82e-29	1.66e-25

Globin Delta	HBD	-12.87	-6.05	4.37e-25	1.52e-21

APX/Shroom Family	SHROOM4	-17.84	-5.37	6.66e-20	1.93e-16

Ataxia/Rad3	ATR	-15.71	4.57	6.80e-17	1.69e-13

Pseudogene	RP11-431J24.4	-21.08	6.1	9.66e-17	2.10e-13

Trub sudouridine synthase	TRUB1	-19.14	4.83	6.43e-16	1.24e-12

Pseudogene	CTD-2165H16.1	-18.82	4.71	1.48e-15	2.58e-12

The 4 most abundant globin transcripts are in the top five of differentially down-regulated transcripts (^1 ^Log Concentration - Overall concentration for a tag across the two groups compared., ^2 ^Log Fold-change - The log-fold change difference for the counts between the groups compared.).

To examine why 83 non-globin transcripts were reduced by the globin reduction procedure, we examined possible non-target specific hybridization of the globin oligos. Using the sequences of the ten biotinylated oligonucleotides from the globin reduction kit we performed a blastn search against the human mRNA reference genome but only the 4 globin transcripts that were found to be most significantly decreased in our globin-reduced samples showed significant hits in blastn. Moreover, oligonucleotide sequences were analyzed with RNAhybrid, an online tool for determining the minimum free hybridization energy between a long and a short RNA [23] but no conclusive evidence was found for non-specific hybridization between oligonucleotides from the globin reduction kit and the 83 non-globin transcripts that were reduced in our globin-reduced samples. Finally we wanted to investigate if any of the 83 transcripts could have been co-captured with the globin transcripts. Hence we investigated whether there was sequence homology between the 83 top reduced transcripts and the globin transcripts but there was no indication of co-capturing based on sequence homology. Furthermore, more than half the transcripts reported as non-specifically detected at lower levels were derived from pseudogenes or expressed at very low levels.

### Transcripts detected in globin-reduced samples only

**Figure 3:**
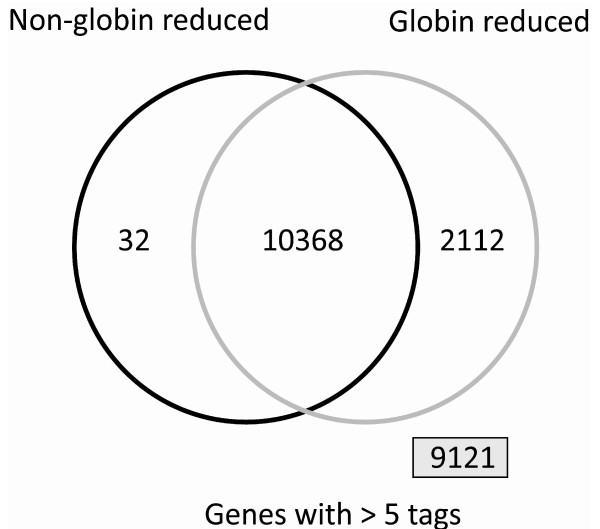
**Venn diagram displaying the number of transcripts in at least 5 out of 6 samples at a level of 5 tags or more**. Reducing the globin transcripts increases the number of transcripts that can be reliably detected (2112 genes). 9121 genes were not consistently expressed in either group.

From the total number of 21,633 transcripts discovered in our SAGE experiments, 10,368 were expressed above the threshold of at least 5 tags in 5 out of the 6 samples in both the globin reduced and non-reduced group. We identified 2,112 transcripts in the globin-reduced samples that were not expressed above the threshold in the non-reduced samples and 32 transcripts in the non-globin reduced samples that could not be detected in the globin-reduced samples (see Figure 3). Additionally, 9,121 transcripts were detected in globin-reduced samples only but did not reach the threshold (low abundance transcripts). Increasing the threshold tag number resulted in a higher number of genes detected by the globin reduced samples while the number of genes only detected in non-reduced samples remained approximately the same. Furthermore increasing the threshold resulted in less genes identified by both groups.

### Functional enrichment and gene biotype analysis

For the 2,112 transcripts identified in the globin reduced samples only, we performed gene ontology (GO) term enrichment analysis using the online DAVID Bioinformatics Resources 6.7 [24] (see Table 3). The GO terms that were most significantly enriched were "metal and ion binding" and "DNA binding", the latter including genes encoding for proteins with roles in transcription and regulation of transcription. We also performed the same enrichment analysis for the 9,121 transcripts that did not reach the more stringent threshold. This group of genes also showed significant enrichment for metal and ion binding proteins. Additionally, we observed enrichment in transcripts coding for cell surface receptors signal transduction proteins, G-protein coupled receptors and proteins involved in neurological processes. Since these transcripts are present in low copy numbers in our samples, these transcripts would benefit from globin reduction and even greater depth of sequencing.

**Table 3:** GO term enrichment analysis using DAVID

GO:	Term	Count	% of genes	*P *value
MF	Ion binding	383	23.7	7.90e-6

MF	Cation binding	380	23.6	4.30e-6

MF	Metal ion binding	379	16.4	2.20e-6

MF	Transition metal ion binding	272	16.9	9.80e-7

MF	Zinc ion binding	229	14.2	3.50e-6

MF	DNA binding	214	13.3	9.50e-4

BP	Regulation of transcription	214	13.3	8.40e-2

CC	Intracellular non membrane bound organelle	210	13.0	1.30e-2

BP	Transcription	183	11.3	2.50e-2

CC	Plasma membrane part	176	10.9	3.70e-2

Top 10 Gene Ontology terms found enriched by the DAVID online analysis tool in the 2,112 transcripts uniquely found in the globin reduced samples. *P*-value reflects the *P*-value from the hypergeometric test. CC-Cellular Component, BP-Biological process, MF-Molecular function.

**Figure 4:**
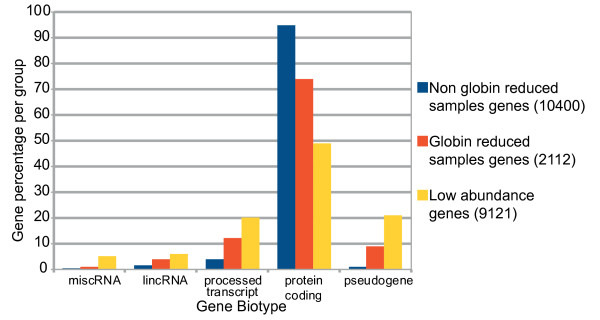
**Gene biotype distribution in transcripts with and without globin reduction**. Percentage of identified transcripts in the non-globin reduced and globin-reduced samples; low abundance transcripts consist of transcripts detected at levels too low to be assigned to any of the two previous groups. The 9121 low abundance transcripts contain relatively more processed transcripts, pseudogenes, large intergenic non coding RNAs (lincRNAs) and miscellaneous RNAs (miscRNAs). We statistically compared the distribution of the number of transcripts over the different categories with a chi-square test and all pairwise comparisons were highly significant (p < 2.2e-16).

There are around 20,000 protein-coding genes that together comprise only 2% of the human genome [25]. However, a large fraction of transcripts map outside known protein coding genes [26] and it is becoming clear that non-protein-coding RNAs play critical roles as transcriptional, post-transcriptional and chromatin-modifying regulators [27]. As such, we were interested in characterizing the gene biotype of the 21,633 transcripts identified in our gene expression analysis (see Figure 4). We analyzed the transcripts according to the groups defined in Figure 3. The majority of transcripts from all three groups were protein coding transcripts. However, there were subtle differences in the identified transcripts. The 2112 transcripts that were uniquely identified in the globin-reduced samples contained slightly elevated numbers of pseudogenes, processed transcripts and large intergenic non-coding RNAs (lincRNAs). The 9121 transcripts whose tag numbers were too low to be consistently ascribed to either of the experimental groups contained relatively more pseudogenes.

### Sequencing Depth vs. Globin Reduction - Genes Discovered

**Figure 5:**
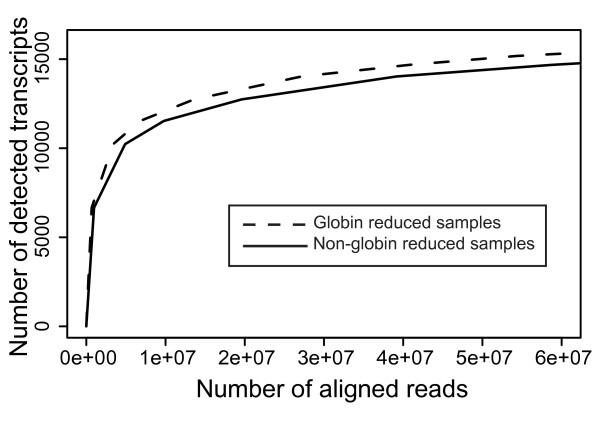
**Number of transcripts detected at increased sampling rate**. In silico analysis using the sum of aligned reads (total number of reads ~60 million) across each group (reduced, non-reduced). A random subset of these aligned reads is then used to find the number of detected transcripts across the two sample groups over the 5 tag, 5 out of 6 samples threshold. The plot shows that at similar sequencing depths, there is a consistently higher number of transcripts detected in the globin reduced samples compared to the non-globin reduced samples.

The previous results showed that by reducing the number of globin transcripts before sequencing, the number of different transcripts that can be identified increases. Next, we investigated how much additional sequencing is required to achieve this effect of globin reduction just by sequencing non-globin reduced samples at a greater depth. For this purpose we compared results from non-globin reduced RNA samples that were sequenced at high and low sequencing depth. At two times higher sequencing depth, the reported alignments as well as the number of identified transcripts for each of the samples increased to the level of the globin reduced samples (Table 4). The number of 13,000 detected transcripts in globin-reduced samples is achieved at a sequencing depth of ~15 million reads while the same number of transcripts is detected at a sequencing depth of ~25 million reads in the non-globin reduced samples. *In silico *analysis was performed to determine at which sampling rate the threshold of at least 5 tags in 5 out the 6 samples was reached. The difference in detected transcripts between the reduced and non-reduced samples at any given sequencing depth remained equal, indicating that even at a very large numbers of reads (> 60 million reads) whole blood samples are not sequenced to saturation (see Figure 5).

**Table 4:** Number of transcripts identified across different sequencing depths

Group	Non-reduced				Reduced	
**Samples**	**Sample1** **Low**	**Sample2** **Low**	**Sample1** **High**	**Sample2** **High**	**Sample1**	**Sample2**

Total number of sequences	7.8^.^10^6^	11.6^.^10^6^	29.2^.^10^6^	32.0^.^10^6^	16.5^.^10^6^	15.9^.^10^6^

Reported Alignments	4.7^.^10^6^	5.5^.^10^6^	23.6^.^10^6^	25.6^.^10^6^	9.8^.^10^6^	9.4^.^10^6^

Transcripts Detected	9,823	9,003	13,085	12,738	13,302	12,831

Two non-globin-reduced samples were sequenced twice, at low and high sequencing depths, and compared to the sequencing results from the same samples with reduction of the globin transcripts.

## Discussion

We performed globin transcript reduction on RNA from human peripheral blood to investigate if this improved the sensitivity of SAGE digital gene expression profiling. In the non-reduced samples, globin percentages were highly variable between samples and ranged from 52% to 76%. These numbers match previous reports for globin transcript abundances in blood [17]. The globin reduction process with biotinylated oligonucleotides complementary to globin transcripts was successful since there was a > 99.6% reduction in globin transcripts. This was consistent with previous studies [28]. We observed that after globin reduction there was a slight decrease in total RNA quality as well as an RNA yield loss of 5-9%. The slight reduction in RNA quality and yield had no effect on the quality of the data. From the 21,633 transcripts that were detected across all 12 samples, 11,633 transcripts were detected at significantly higher levels in the 6 globin reduced samples, while only 87 transcripts were reported as detected at significantly lower levels. We can not explain why there were transcripts, other than the globin transcripts, found to be expressed at lower levels in the globin reduced samples. In silico analysis showed that this was not likely due to cross hybridization with the globin transcripts or with the globin oligonucleotides from the globin reduction kit.

We detected robust expression of 2112 transcripts in globin reduced samples that could not be detected in non-globin reduced samples. This is similar to what has been previously reported using classical microarray gene expression platforms [17]. The important advantage of more detectable transcripts is that there will be increased statistical power to detect differences between samples. Furthermore, this increased number of detected transcripts after globin reduction provides more information about low abundance transcripts that could give new insights into the underlying disease mechanism.

The reduction of globin transcripts provides a practical and reproducible method for improving the number of transcripts detected in human peripheral whole blood samples, and reduces required sequencing capacity by a factor two. Deep SAGE is a technique that detects short tags of 21-22 base pairs representing specific transcripts. For this reason the data complexity is lower compared to other digital gene expression profiling techniques such as whole transcriptome sequencing (RNA-seq). For RNA-seq, where the aim is to also identify all the transcript isoforms, saturation is definitely not reached at 25 million reads, which would make globin transcript reduction in blood even more advantageous for RNA-seq.

## Conclusions

The reduction of globin transcripts together with whole blood collection methods, such as the PAX system, constitutes a reproducible and reliable method that increases the number of transcripts detected in next generation sequencing-based gene expression profiling. This will increase the statistical power to detect disease relevant signatures in patient-control studies.

## Methods

### Blood Collection & RNA isolation

Samples from six healthy individuals of Caucasian decent (4 females, 2 males) aged from 39 to 70 years old were collected after informed consent and with ethical approval. Whole blood was drawn into PAX gene tubes (Qiagen, Venlo, The Netherlands) and inverted 10 times. Samples were allowed to equilibrate at room temperature for 2 hours, placed at -20°C overnight and stored at -80°C until further processing. Before total RNA extraction, samples were thawed overnight at 4°C and total RNA was isolated using the PAX RNA isolation kit following the manufacturer's instructions, including DNAse treatment. RNA quality and the RIN values were determined using the RNA Nano LabChip assay (Agilent Technologies, Santa Clara, CA, USA). The concentration of each sample was validated using the Nanodrop spectrophotometer (NanoDrop Technologies, Wilmington, DE, USA).

### Globin Reduction

From each sample, 1.5 μg of total RNA was treated using the GLOBINclear™ Human Kit (Ambion, Austin, TX, USA) according the manufacturer's instructions. The kit contains 10 globin sequence specific biotinylated oligonucleotides http://www.freepatentsonline.com/y2006/0257902.html. Transcripts hybridized to the oligonucleotides are removed through streptavidin magnetic beads. Concentration and quality of the samples were checked as described above.

### Quantitative Real-Time PCR

cDNA was synthesized from 1 μg of total RNA using the Transcriptor First Strand cDNA Synthesis Kit (Roche, Mannheim, Germany) with Random Hexamer primers at 50°C for 1 hour. The primer sequences designed to target genes of interest and the reference gene *ACTB *are described in Additional file 4. The qPCR was performed using 1 μl of 4x diluted cDNA, 2x FastStart Universal SYBR Green Master mix (Roche), 2.5 pmol forward primer,2.5 pmol reverse primer and PCR grade water to a total volume of 10 μl. The reaction was performed using the LightCycler 480 (Roche) with initial denaturation 10 min. at 95°C, followed by 45 cycles of 10 sec. denaturation at 95°C, 30 sec. annealing at 60°C and 20 sec. elongation at 72°C and final elongation was performed 5 min. at 72°C. This was followed by melting curve analysis from 60°C to 98°C with a ramp rate of 0.02°C per sec. The primer efficiencies and relative expression of the transcript levels was determined using LinRegPCR_v11.3[29].

### SAGE library production

SAGE libraries were produced according to the Illumina protocol [6]. In short, after hybridization of 1 μg total RNA to polydT magnetic beads (Dynabeads, Invitrogen Life Technologies, Carlsbad, CA, USA), first and second strand synthesis was performed. The beads attached to the double stranded DNA were digested with NlaIII restriction endonuclease to produce short double stranded constructs starting at the most 3' CATG of the transcript. After ligation of the GEX adapter 1, the construct was digested with MmeI to create a 21 base pair fragment downstream of the GEX adapter 1. This fragment was then ligated to GEX adapter 2 to complete the cassette. The adaptor ligated constructs were amplified by 15 cycles of PCR and the products were loaded on 6% Novex TBE precast acrylamide gels (Invitrogen). The 96 base pair band corresponding to the NlaIII construct was excised and purified from the gel slice using the soak and crush method followed by ethanol precipitation. Sample quality was checked on a DNA 1000 Lab-on-a-Chip (Agilent). Sequencing was performed at the Leiden Genome Technology Center on Illumina GA2 sequencer (Illumina, San Diego, CA, USA). Purified samples were diluted to 10 nM and loaded on a single lane of the flow cell where, after cluster amplification, samples were put through an ultra short 18 cycle sequencing run.

### Sequence processing

Illumina Pipeline Software version 1.5 was used for data sequence processing. The FASTQ files were analysed using the open source GAPSS_B(v2) pipeline http://www.lgtc.nl\GAPSS. All sequences were trimmed to 17 base pairs to remove the first lower quality base pair from the 3' end of the sequences. After trimming, the NlaIII recognition site (CATG) was added to the 5' end of the sequence to create the complete 21-22mer nucleotide sequences. Sequences were aligned using the Bowtie short read aligner (version 0.12.7) against the UCSC hg19 reference genome, allowing for a maximum of one mismatch and a maximum of two possible positions in the genome (options: -k 1 -m 2 -n 1 --best --strata -solexa1.3-quals).

A custom Perl script was used to create reference region files from the SAGE region files that were composed of the overlapping tags from all samples. A second Perl script was then used to link all individual region files to the reference region file, reporting the number of tags in each individual region of the reference region file. Finally, the reference region file was annotated with transcript information using BIOMART (Ensembl build 60). For all reported downstream statistical and biological analysis only sequences aligned to known exons were used. Analyses were performed at the gene level, and in case of multiple SAGE tags per gene, e.g. as a consequence of alternative polyadenylation, tags were summed. The data discussed in this publication have been deposited in NCBI's Gene Expression Omnibus and are accessible through GEO Series accession number GSE33701 http://www.ncbi.nlm.nih.gov/geo/query/acc.cgi?acc=GSE33701.

### Statistical Analysis

To identify genes detected at lower or higher levels, data files containing the count expression data were analyzed using the edgeR package (version 2.0.5) [30,31] in R (version 2.12.0). Data was normalized creating libraries of equal size (11 million tags). To determine differences in detection levels between the two groups an exact test for the negative binomial distribution was used. The *P*-values were adjusted for multiple testing using the Benjamini and Hochberg's approach for controlling the false discovery rate (FDR) and genes were considered to be different between reduced and non-reduced samples when the *P *value was less than 0.01. For pathway enrichment analysis, the online tool DAVID was used. To identify gene biotypes, Ensembl genes IDs were uploaded to the BioMart site and supplied gene biotype attributes (ENSEMBL build 60, Homo sapiens genome reference GRCH37.p2).

To examine if transcripts are uniquely and consistently identified in the globin reduced or non-reduced samples only, we applied a tag count threshold of 5 tags or more and this threshold had to be met in 5 out of the 6 samples in the respective group.

## Authors' contributions

AM carried out the sample processing, sequencing, statistical analysis, and drafted the manuscript. JTDD participated in the design of the study and helped to draft the manuscript. GJBVO participated in the design of the study and helped to draft the manuscript PACTH participated in the design of the study, the statistical analysis and helped to draft the manuscript. WMCVRM conceived the study, and participated in its design and coordination, and helped to draft the manuscript. All authors have read and approved the final manuscript.

## Supplementary Material

Additional file 1 **Hemoglobin qPCR results**. qPCR results for the hemoglobins before and after globin reduction. 4 non reduced and 4 reduced samples were loaded in triplicates and beta actin (ACTB) was used as a reference gene. Click here for file

Additional file 2 **Correlation matrix for the Globin reduced samples**. Upper panels represent correlation values of log transformed data with stars depicting significance. Lower panels contain scatter plots of log transformed globin reduced samples. Click here for file

Additional file 3 **List of 87 down-regulated genes**. List of 87 genes detected at significantly lower levels in the globin reduced samples. Click here for file

Additional file 4 **qPCR primer Sequences**. Primer sequences used for qPCR validation of HBA, HBB transcript levels. Click here for file
