# Supplementary material for: Increased sensitivity of next generation sequencing-based expression profiling after globin reduction in human blood RNA
Source: BMC Genomics. 2012 Jan 18;13:28. doi: 10.1186/1471-2164-13-28 (PMC3275489; doi:10.1186/1471-2164-13-28)
Supplement: Additional file 1 — Hemoglobin qPCR results. qPCR results for the hemoglobins before and after globin reduction. 4 non reduced and 4 reduced samples were loaded in triplicates and beta actin (ACTB) was used as a reference gene. [file 1471-2164-13-28-S1.PDF]

A

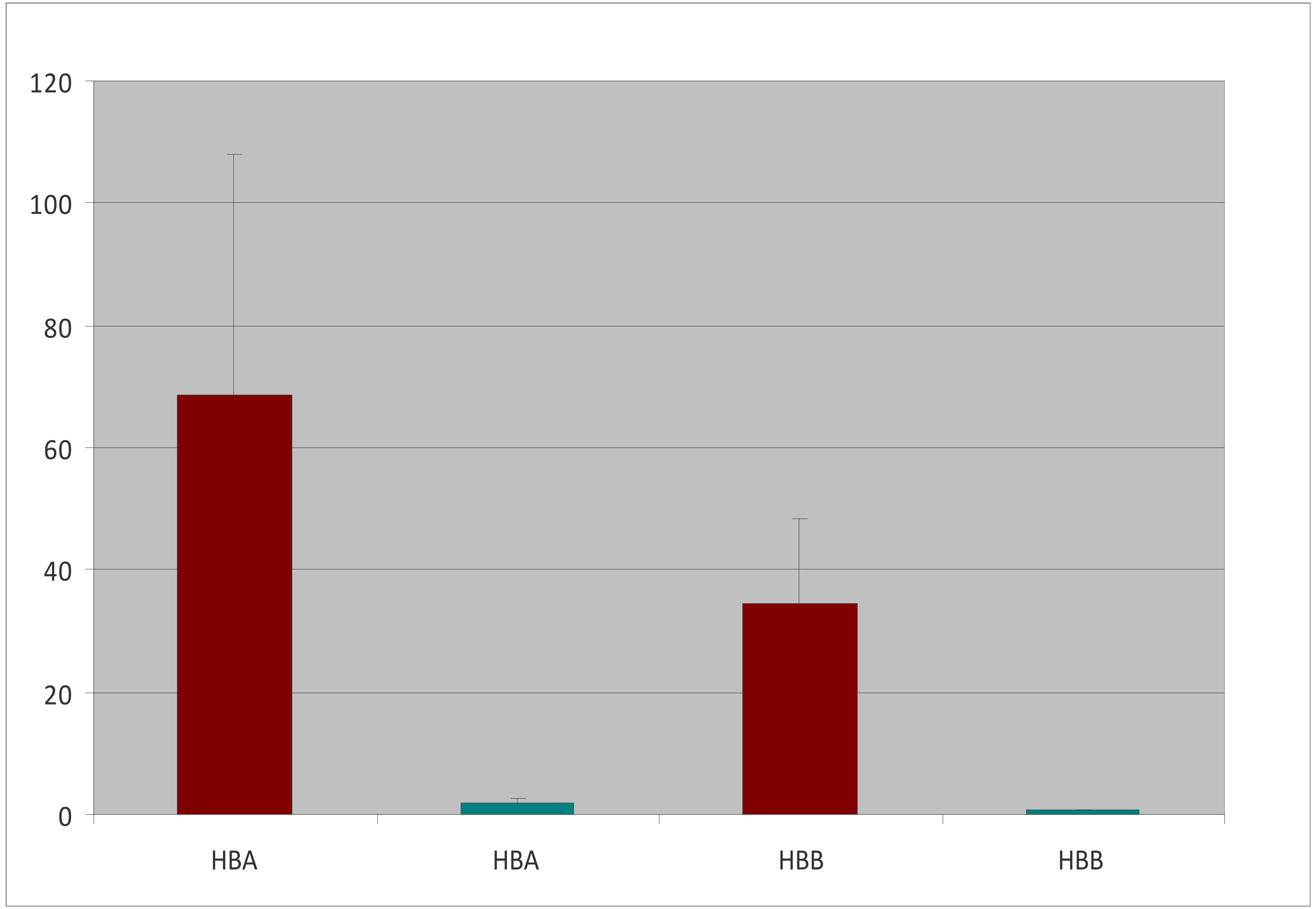

B

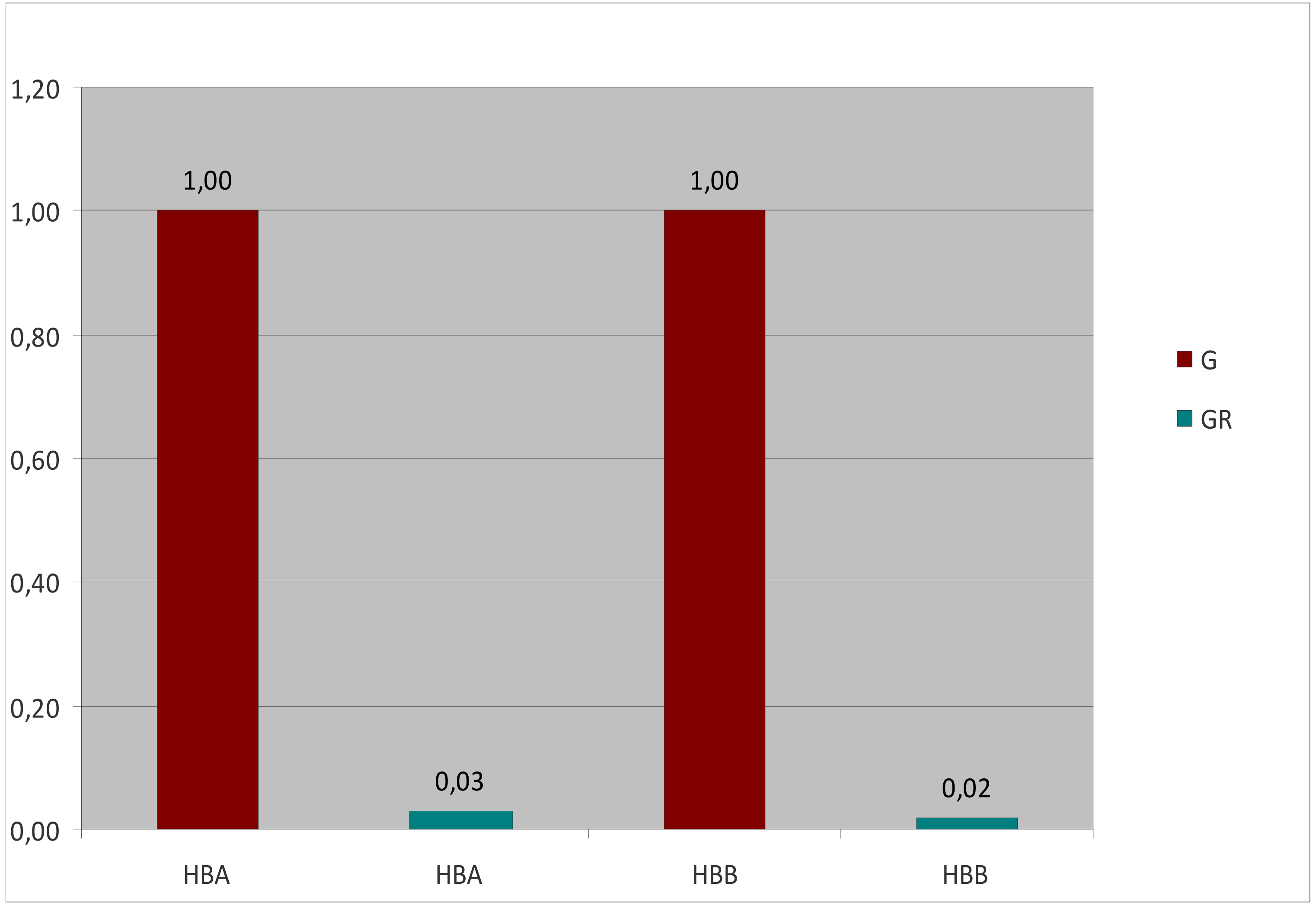

Additional file 1. qPCR results showing relative (A) and absolute (B) hemoglobin alpha (HBA) and hemoglobin beta (HBB) levels before (G) and after globin reduction (GR). Results represent the biological average of 4 samples.
