# Supplementary figures and images for: Increased sensitivity of next generation sequencing-based expression profiling after globin reduction in human blood RNA
Source: BMC Genomics. 2012 Jan 18;13:28. doi: 10.1186/1471-2164-13-28 (PMC3275489; doi:10.1186/1471-2164-13-28)

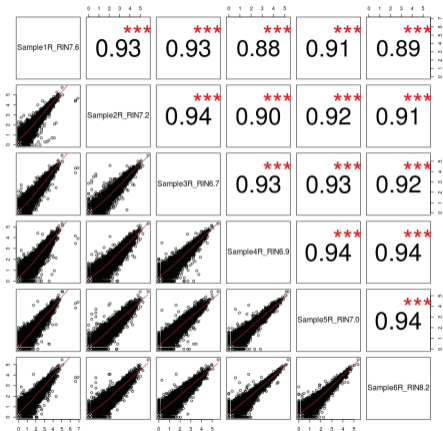

Supplement: Additional file 2 — Correlation matrix for the Globin reduced samples. Upper panels represent correlation values of log transformed data with stars depicting significance. Lower panels contain scatter plots of log transformed globin reduced samples. [file 1471-2164-13-28-S2.PDF]
