# Supplementary material for: Increased sensitivity of next generation sequencing-based expression profiling after globin reduction in human blood RNA
Source: BMC Genomics. 2012 Jan 18;13:28. doi: 10.1186/1471-2164-13-28 (PMC3275489; doi:10.1186/1471-2164-13-28)
Supplement: Additional file 3 — List of 87 down-regulated genes. List of 87 genes detected at significantly lower levels in the globin reduced samples. [file 1471-2164-13-28-S3.PDF]

Ensembl ID	Gene Name
ENSG00000242667	RP11-745A24.2
ENSG00000228955	RP11-452J6.2
ENSG00000240861	RP11-572F4.1
ENSG00000092445	TYRO3
ENSG00000177452	RP4-597J3.1
ENSG00000230228	RP11-4M23.4
ENSG00000231634	RP4-590F24.2
ENSG00000242199	RP11-71H17.1
ENSG00000226259	GTF2H2
ENSG00000148795	CYP17A1
ENSG00000202029	U6
ENSG00000252678	RNaseP_nuc
ENSG00000216548	RP11-471B18.1
ENSG00000216368	RP3-486D24.1
ENSG00000124721	DNAH8
ENSG00000233522	AC007241.4
ENSG00000187682	ERAS
ENSG00000104415	WISP1
ENSG00000125148	MT2A
ENSG00000248907	RP11-627H22.4
ENSG00000235169	RP1-286D6.2
ENSG00000229608	AL049757.2
ENSG00000213752	CTA-268H5.9
ENSG00000233028	RP4-725G10.4
ENSG00000240376	RP11-36C20.1
ENSG00000213465	ARL2
ENSG00000249264	AC004066.1
ENSG00000177212	OR2T33
ENSG00000233193	AC005324.7
ENSG00000243829	CTB-33G10.1
ENSG00000214914	RPL23AP3
ENSG00000237347	AC004461.4
ENSG00000189238	AC069235.1
ENSG00000112697	TMEM30A
ENSG00000144366	GULP1
ENSG00000184319	AC002055.4
ENSG00000205106	AC044839.1
ENSG00000188886	ASTL
ENSG00000187809	AC012555.1
ENSG00000162076	FLYWCH2
ENSG00000104969	SGTA
ENSG00000160972	PPP1R16A
ENSG00000186998	EMID1
ENSG00000158856	EPB49
ENSG00000226243	RPL37AP1
ENSG00000132688	NES
ENSG00000242215	RP11-513M16.2

ENSG00000183663	RP11-44N17.1
ENSG00000147573	TRIM55
ENSG00000213798	AC004129.9
ENSG00000203362	RP3-337H4.8
ENSG00000116774	OLFML3
ENSG00000107147	KCNT1
ENSG00000229770	CTA-796E4.4
ENSG00000223828	RP11-64I24.1
ENSG00000242071	RP11-880O3.1
ENSG00000230370	AC005007.3
ENSG00000173464	RNASE11
ENSG00000131469	RPL27
ENSG00000247306	AL160398.1
ENSG00000164418	GRIK2
ENSG00000225478	RP1-8B22.2
ENSG00000130204	TOMM40
ENSG00000188425	NANOS2
ENSG00000102230	PCYT1B
ENSG00000165566	FAM123A
ENSG00000187893	CXorf25
ENSG00000188536	HBA2
ENSG00000175054	ATR
ENSG00000215313	RP11-431J24.4
ENSG00000105640	RPL18A
ENSG00000213399	AC022210.2
ENSG00000140798	ABCC12
ENSG00000243023	UBA52P3
ENSG00000244734	HBB
ENSG00000223609	HBD
ENSG00000198923	AC011611.2
ENSG00000165832	TRUB1
ENSG00000250182	CTD-2165H16.1
ENSG00000105398	SULT2A1
ENSG00000232873	AC046143.6
ENSG00000130656	HBZ
ENSG00000158352	SHROOM4
ENSG00000242602	CTD-2339M3.1
ENSG00000241376	AL606830.1
ENSG00000206172	HBA1
ENSG00000226970	RP11-82H13.2
