# Supplementary material for: Increased sensitivity of next generation sequencing-based expression profiling after globin reduction in human blood RNA
Source: BMC Genomics. 2012 Jan 18;13:28. doi: 10.1186/1471-2164-13-28 (PMC3275489; doi:10.1186/1471-2164-13-28)
Supplement: Additional file 4 — qPCR primer Sequences. Primer sequences used for qPCR validation of HBA, HBB transcript levels. [file 1471-2164-13-28-S4.PDF]

Primer Sequences - qPCR

HBA-FWD	ACTCTTCTGGTCCCCACAGA	75bp product
HBA-REV	GCCTTGACGTTGGTCTTGTC	
HBB-FWD	TTGAGTCCTTTGGGGATCTG	75bp product
HBB-REV	CTTTCTTGCCATGAGCCTTC	
BACTIN-FWD	GGCATCCTCACCCTGAAGTA	82bp product
BACTIN-REV	AGGTGTGGTGCCAGATTTTC	
